# Tumor-Induced Inflammatory Cytokines and the Emerging Diagnostic Devices for Cancer Detection and Prognosis

**DOI:** 10.3389/fonc.2021.692142

**Published:** 2021-07-07

**Authors:** Apriliana E. R. Kartikasari, Cesar S. Huertas, Arnan Mitchell, Magdalena Plebanski

**Affiliations:** ^1^ Translational Immunology and Nanotechnology Research Program, School of Health and Biomedical Sciences, RMIT University, Bundoora, VIC, Australia; ^2^ Integrated Photonics and Applications Centre (InPAC), School of Engineering, RMIT University, Melbourne, VIC, Australia

**Keywords:** cancer, inflammation, tumor microenvironment, cytokines, diagnosis, prognosis, biomarkers, point of care

## Abstract

Chronic inflammation generated by the tumor microenvironment is known to drive cancer initiation, proliferation, progression, metastasis, and therapeutic resistance. The tumor microenvironment promotes the secretion of diverse cytokines, in different types and stages of cancers. These cytokines may inhibit tumor development but alternatively may contribute to chronic inflammation that supports tumor growth in both autocrine and paracrine manners and have been linked to poor cancer outcomes. Such distinct sets of cytokines from the tumor microenvironment can be detected in the circulation and are thus potentially useful as biomarkers to detect cancers, predict disease outcomes and manage therapeutic choices. Indeed, analyses of circulating cytokines in combination with cancer-specific biomarkers have been proposed to simplify and improve cancer detection and prognosis, especially from minimally-invasive liquid biopsies, such as blood. Additionally, the cytokine signaling signatures of the peripheral immune cells, even from patients with localized tumors, are recently found altered in cancer, and may also prove applicable as cancer biomarkers. Here we review cytokines induced by the tumor microenvironment, their roles in various stages of cancer development, and their potential use in diagnostics and prognostics. We further discuss the established and emerging diagnostic approaches that can be used to detect cancers from liquid biopsies, and additionally the technological advancement required for their use in clinical settings.

## Background: Tumor Microenvironment, Inflammation, and Cytokines

The initiation and subsequent development of a tumor into a metastatic state are not only driven by genetic or epigenetic changes but also greatly determined by the action of the tumor microenvironment (TME) (1). Besides tumor cells, TME commonly contains other non-transformed cells, including the stromal cells such as fibroblasts, mesenchymal stem cells, endothelial cells, and pericytes, and also the immune cells such as macrophages and lymphocytes. The dynamic interactions between tumor cells and the non-transformed cells are key in determining the progression of cancer, as these could either suppress or promote cancer initiation, growth, migration, and metastasis, as well as cancer recurrence and drug resistance (2), such as stromal cell cues that help cancer growth and invasion, and endothelial cell responses that promote the generation of new blood vessels to the cancer site.

In the TME, certain immune cell infiltrates are correlated with improved cancer outcomes, however, some studies show that unresolved host immune reactivity could lead to chronic inflammation and promotes tumor growth. The unresolved host immune reactivity is mainly due to the dynamic interactions between tumor cells and the recruited immune and other non-transformed cells, mediated by cytokines. Cytokines can instruct biological processes of cells including growth, differentiation, proliferation, and migration. Cytokines are small proteins of up to 70 kDa (3). Based on their structure and function, they have been classified in distinct superfamilies including interferons (INFs), interleukins (ILs), tumor necrosis factors (TNFs), transforming growth factors (TGFs), chemotactic cytokines (chemokines), and colony-stimulating factors (CSFs) (4). Individual cytokines however have their specific spatiotemporal functions and exert their effects through autocrine and paracrine mechanisms (5). The tumor cells are known to secrete cytokines that can both in autocrine fashion generate a forward-feedback loop to stimulate self-proliferation, expansion, and drug resistance, and in paracrine fashion induce recruitment, activation, and differentiation of other cells in the TME, such as IL-6, IL-8 and even VEGF (6–8). The cytokines commonly alert immune cells to the presence of infections and tissue damage, however persistent cytokine production at a certain body site could, in turn, stimulate immune cells to secrete more cytokines that work in both autocrine and paracrine manners leading to a chronic inflammation state that promotes cancer growth (9). The inflammatory response towards tissue damage and infections shares molecular and signaling pathways with carcinogeneses, such as induction of cell proliferation and angiogenesis (10). In the TME, cytokines may build a tumor-supportive immune microenvironment, that suppresses anti-tumor immunity and exerts direct tumor-promoting signals (11). This process not only happens locally, but cytokines from TME can also exert their biological actions distantly *via* circulation, supporting metastasis. This chronic inflammatory condition can be exacerbated by other conditions such as obesity, sedentary lifestyle, cigarette smoking, alcohol consumption, and chronic infections, that promote low-grade systemic inflammation (12). The systemic inflammation in turn accelerates cancer progression by changing the dynamic of the TME and inducing a further cancer-supportive environment (9).

**Figure 1:**
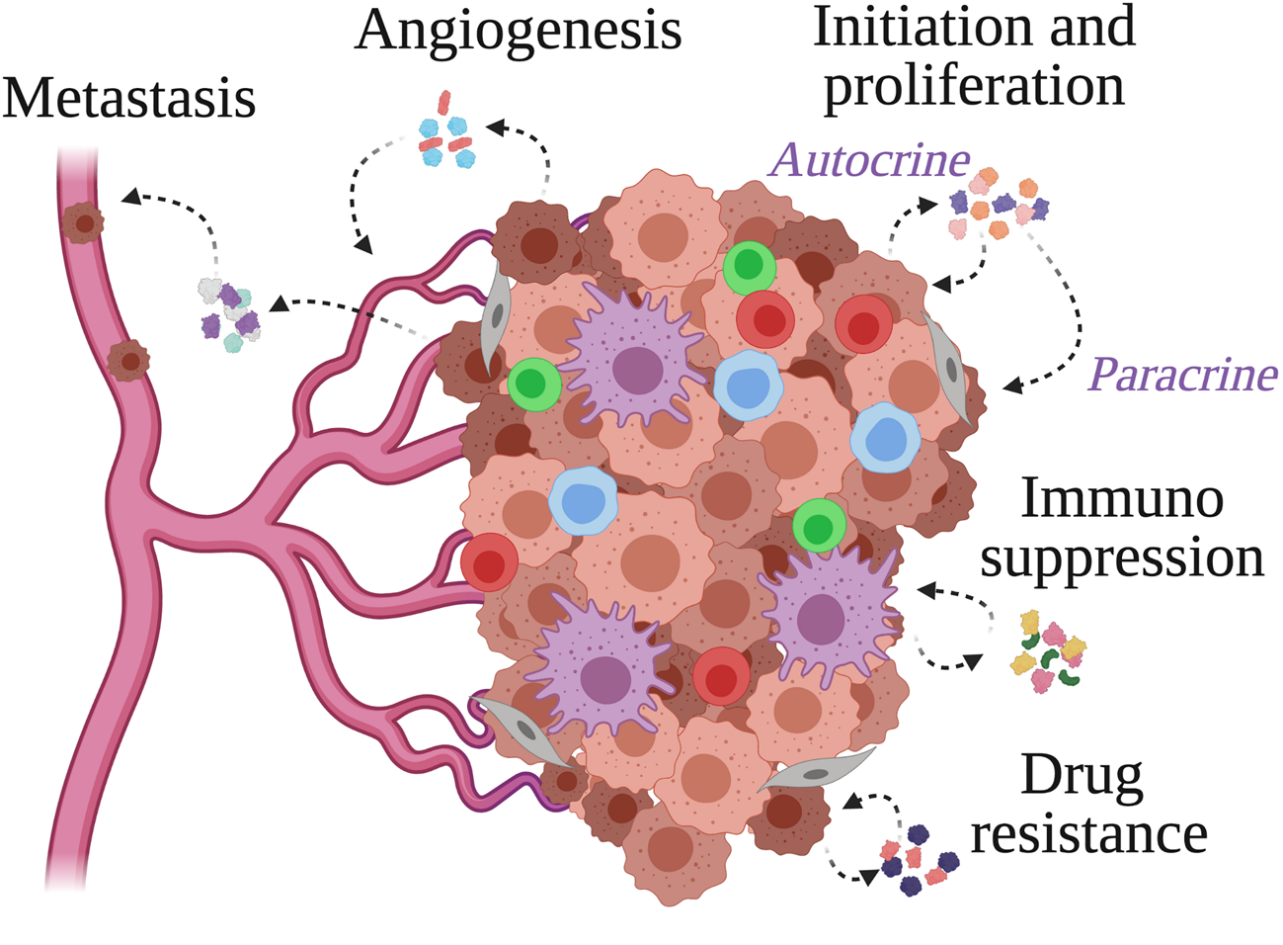
Cytokine actions within the tumor microenvironment. Cancer cells, stromal cells and immune cells populate the tumor microenvironment and secrete cytokines to facilitate the events supporting tumor growth. These include tumor cell initiation and proliferation, immunosuppression, angiogenesis, metastasis, and resistance to anti-cancer drugs.

The set of cytokines involved in the pro-cancer TME inflammation at a given time during cancer development could be specific as various cells may release distinct pathogenic cytokines at a specific time of the disease progression (**Figure 1**). The production of cytokines is regulated at transcriptional and post-transcriptional levels, and the downstream signaling by the cytokines is dependent on the availability of the cytokine receptors that are also subject to both transcriptional and post-transcriptional regulation. In cancer cells often, cytokines and the receptors are overexpressed, not only by a direct increase in transcription but also due to abnormal RNA stabilization that promotes excessive protein expression (13), preventing the resolution of inflammation that promotes cancer growth (14). Besides promoting cell proliferation, the action of cytokines in promoting cancer development includes antagonizing anti-tumor immune response, recruiting tumor-supportive stromal cells and immune-suppressive cells, inducing angiogenesis and metastasis, and altering the responses to therapeutic agents (10, 15) (**Figure 1**). On the other hand, cytokine production can be triggered by oncogenic transformation, metabolic alteration, cell death, hypoxia, and the use of anti-cancer drugs (10). This thus provides an opportunity to use cytokines and the associated cytokine receptors as diagnostic and prognostic markers to detect and monitor cancer development respectively.

In the course of chronic inflammation, multiple cytokines come and go and each of them serves redundant tasks (16). A single cytokine measurement may only be useful as a confirmation marker of the presence of a recognized disease. Profiling a set of cytokines instead could be more informative to help define the presence and further the severity of complex diseases like cancer (16, 17). The tumor cells themselves may secrete cancer-specific proteins that often support cancer growth and/or metastasis. These circulating cancer-derived proteins, although are often not specific enough, have been proposed as biomarkers to indicate the presence of cancers. Other approaches for cancer diagnosis that are used at clinics include imaging-based diagnostics that would require exclusive instruments and phenotypic determination of tumor biopsy that would be invasive. Combining measurements of cancer-specific proteins with the circulating cytokines from liquid biopsies such as blood, could help accurately detect cancer, and may further determine its stage. This approach of cancer detection is not only minimally invasive and cost-effective but also could be easily accessible as various analytical technologies are available without the requirement of exclusive instruments. Here we will discuss cytokines that are involved in cancer development at different stages. We will also discuss strategies involving cytokines that can be used for cancer detection or prognosis. Additionally, we will discuss the current and emerging diagnostic tools involving cytokine detection for cancer and their potential technological challenges. The development of diagnostic tools that are simple and affordable will enable cancer screening and monitoring at the point of care especially in places with a high cancer burden.

## Inflammation Induces Genetic and Epigenetic Changes Promoting Development of Tumor

**Figure 2:**
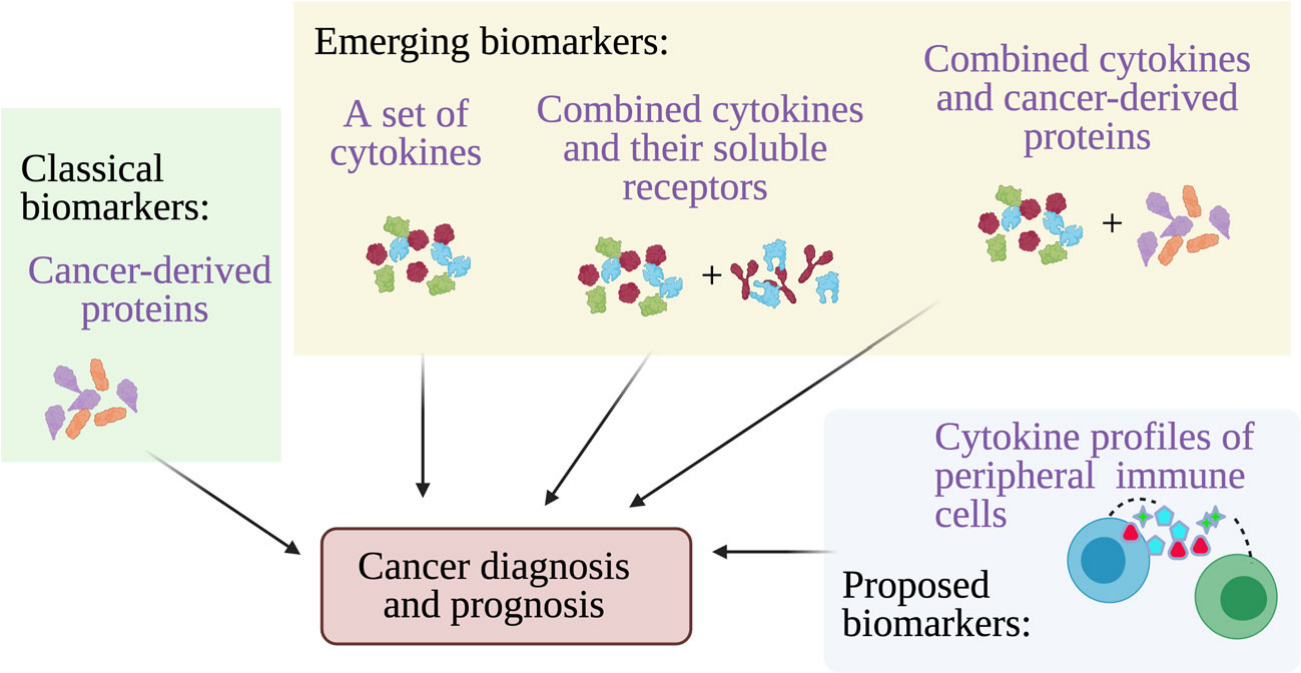
Cytokines for cancer diagnosis and prognosis. Multiple cancer-derived proteins have been established and used at clinics as biomarkers to detect cancer from blood, however other approaches including detection of a set of cytokines, combining cytokines and their soluble receptors, and combining cytokines and cancer proteins may provide better accuracy for cancer diagnosis and prognosis. A highly accurate alternative cancer biomarkers have been proposed which is the cytokine secretion profile of the circulating peripheral immune cells.

The initiation and progression of cancers require activation of the oncogenic pathways and on the contrary inactivation of the tumor-suppressive pathways. The alteration of these pathways is mainly due to the accumulation of genetic mutations and/or epigenetic changes that activate or silence genes related to the oncogenic or tumor-suppressive pathways respectively. It is well accepted that those genetic mutations and epigenetic changes could be the result of inherent genetic predisposition or extensive exposure to extrinsic mutagens such as carcinogens and radiations. However, a growing body of evidence shows that local chronic inflammation by itself is a potent inducer of genetic mutations and epigenetic modifications, without the presence of genetic predisposition nor extrinsic mutagens (9). The inflammatory response predisposes cancer initiation and progression at the local tissue. Inflammatory cytokines produced in the local inflammatory site are capable of promoting the production of reactive oxygen and nitrogen species that in turn damage DNA and promote DNA mutations. They can also directly alter the epigenome of the cells, including DNA methylation, histone modifications, and regulatory RNA expressions that in turn activate the oncogenic pathways and inactivate the tumor-suppressive pathways (18). On the other hand, the inflammatory cytokines themselves are subject to epigenetic (19) and post-transcriptional dysregulations (13) in cancer. As an epigenetic signature is reversible, remodeling of the epigenetic signatures of the inflammatory cancer cells could potentially be used as a therapeutic strategy. Indeed, epigenetic remodeling of the loci of inflammation-related genes including cytokines has been shown recently to suppress cancer growth and metastasis (20). Additionally, understanding the dysregulated post-transcriptional events in cancer cells could also lead to therapeutic targets (13). Since alteration in the levels of cytokines can change cancer growth, detection of the cytokines themselves could be useful for diagnosis, determining therapy of choice, and monitoring the progression of the disease (**Figure 2**).

## Cytokines in Tumor Growth

Within the TME, cytokines mediate cell-to-cell interactions to promote tumor growth. It is now appreciated that tumor-induced cytokine production and inflammation in the TME promote and accelerate cancer development (9, 14). The first evidence of cytokine role in tumor growth is coming from a study using a model of colitis-associated cancer, whereby inactivation of NF-kB in myeloid cells diminish the expression of cytokines and reduces tumor size (21). NF-kB is the master regulator of many cytokines that involve in the induction of cell growth, proliferation, and cell recruitment, which all shape the TME (22). Signaling by the inflammatory cytokines induced by NF-kB including IL-1, IL-6, TNF, IL-8, IL-17, IFN-γ, and CCL-5 among others (23) promotes tumor growth by induction of cell proliferation (24) in both autocrine and paracrine manners (25).The local senescence cells secrete the senescence-associated secretary phenotype (SASP), which includes a vast array of pro-inflammatory cytokines including IL-1α, IL-1β, IL-6, IL-8, CXCL-1, and CXCL-2, that is capable of inducing tumorigenesis in a paracrine fashion (26).

The involvement of cytokines in tumor growth is also seen in KRAS activation. KRAS is the most frequently mutated isoform of the oncogenic RAS, occurring in approximately 20-25% of all human cancers (27). KRAS constitutively activates NF-kB and drives the expression of cytokines that belong to SASP, with the most prominent cytokine being secreted being IL-6 (28). IL-6 plays a significant role in inducing tumor growth as it interacts with the receptor, JAK, to induce STAT-3 activation. STAT-3 then activates oncogenes such as MCL-1 (29) and upregulates genes involved in proliferation such as Cyclin-D1 (30). These activities result in the induction of cancer cell proliferation. IL-6 *via* STAT-3 has been shown to promote tumor cell proliferation in many cancers, including oral squamous cell carcinoma *via* DNA hypomethylation (31), prostate cancer through androgen receptor activation (32), and colon cancer (33). The anti-inflammatory cytokine, IL-10 can paradoxically induce STAT-3 activation (34), which also leads to induction of tumor cell proliferation (35–37). Interestingly, the CMV-derived IL-10 also promotes the proliferation and migration of cancer cells (38, 39), linking chronic infections such as CMV to cancer formation (40).

The p53 tumor suppressor gene plays a central role in the induction of apoptosis, and mutations of the gene have contributed to 50% of all cancer mutations. Several studies have shown that p53 mutants can prolong TNF-induced NF-kB activation, induce SASP and promote the survival and proliferation of tumor cells (41, 42). In the ovarian organoid model, prolonged TNF exposure confers precancerous phenotypes with high expression of cancer markers (43). TNF also induces the formation of cancer stem-like phenotypes in oral squamous cell carcinoma (44, 45). Furthermore, TNF can directly stimulate breast cancer proliferation *via* the positive feedback loop of TNFR-1/NF-kB/STAT-3 (46) as well as be involved in cathepsin C-induced hepatocellular malignancy *via* MAPK signaling pathway (47). Other cytokines including IL-1β (48), IL-8 (49), IL-17 (50) and IL-11 (51) have been extensively reviewed for their involvement in tumor development and progression. In the TME, TNF can promote the secretion of IL-17 (52) and IL-11 (53) by cancer cells, indicating an indirect action of TNF in promoting tumor growth.

The presence of immune cells further complicates the unresolved inflammation state in the TME, in which cancer cells avoid destruction by the immune system and at the same time develop an immunosuppressive environment and stimulate cancer cell proliferation and metastasis. In many oncological conditions as reviewed in (26) and (54), the presence of tumor-associated macrophages (TAMs), tumor-associated neutrophils (TANs), myeloid-derived suppressor cells (MDSC), and regulatory T cells (Tregs) in the TME is associated with poor prognosis and outcome, as they produce IL-10, TGF-β, and prostaglandins that are potent immunosuppressors, suppressing the anti-tumor activities of NK, T and B cells in the TME, allowing survival and proliferation of cancer cells.

## Cytokines in Cancer Metastasis and Invasion

Metastatic disease has caused 90% of cancer-related deaths. Inflammation has been underscored as the cause of the metastatic process (55). The process of metastasis is started by epithelial-to-mesenchymal transition (EMT), whereby the tumor cells lose their cell-to-cell adhesion mediated by E-cadherin, and independently capable of breaking through the tumor membrane and entering the bloodstream, invading the surrounding tissues and further reaching the lymphatics or blood vessels to further metastasize. The inflammatory mediator that strongly promotes EMT is TGF-β. Adding TGF-β induces EMT in diverse epithelial cell cultures *via* the SMAD signaling pathway (56). In collaboration with the canonical and non-canonical Wnt signaling, TGF-β functions in an autocrine manner to maintain the mesenchymal state (57). The cytokines IL-1β, TNF, and IL-6 can further enhance the EMT transition. IL-6 *via* the JAK/STAT pathway has been shown to promote EMT in head and neck cancer (58), while JAK/STAT pathway itself is required for TGF-β. -induced EMT formation in lung cancer (59). Both TNF and IL-6 activate NF-kB that directly promote EMT by upregulating transcription factors involved in EMT (60–62). Moreover, the two cytokines may synergistically enhance TGF-β. signaling by activating multiple pathways (63–66). Recently IL-1β has been shown to promote EMT *via* epigenetic modifications in non-small cell lung cancer (67).

Angiogenesis is crucial for the survival of the tumor colony, as the formed blood vessels will supply nutrients and oxygen to the cancer cells, and later for the spread of the cancer cells. Angiogenesis is induced by several factors including VEGF and FGF (68), IL-8 (49) and TGF-β (69), mainly secreted by cancer cells, but also TAMs, endothelial cells, and fibroblasts in the TME. IL-6 *via* STAT-3 is a potent inducer of the secretion of angiogenesis factors by the cells in the TME (70). Additionally, TNF promotes myeloid to endothelial differentiation required for angiogenesis (71). Similar to angiogenesis, lymphangiogenesis can be generated by cancer cells by the secretion of VEGF to induce the formation of new lymph vessels (72).

Following the formation of blood and lymph vessels, metastatic spread of the cancer cells can occur utilizing these newly formed vessels. The circulating cytokines could help in establishing and accelerating the metastatic capability of cancer cells. For example, IL-17 released by δγ-T cells that is induced by IL-1β secreted by TAMs, promotes metastasis environment in breast cancer (73, 74). Both IL-6 and TNF produced by myeloid cells in the TME can directly stimulate metastasis and invasion (58, 75–78).

## Cytokines Induced Due to Therapy and Resistance to Therapy

Although may not be only originated in the TME, another important type of inflammation is cancer therapy-induced inflammation (14). This type of inflammation also exacerbates the chronic and age-related inflammation that cancer patients already have (79). Various anti-cancer therapies including chemotherapy, radiotherapy, as well as the newly developed biologic therapies and immunotherapies could induce cancer cell death and further potentiate inflammation. The inflammation that induces stronger immune responses could be beneficial for anti-tumor immunity. The use of checkpoint inhibitors to lower the immune system’s self-tolerance has been used to promote stronger immune responses (80). However certain immunostimulatory cytokines may not be beneficial to treat cancer and may unintentionally promote tumor regrowth or lead to immunosuppression (81). In this scenario, dying tumor cells may stimulate the production of cytokines that in turn promote cancer drug resistance.

Multiple drug resistance has been observed when IL-6 is highly expressed by cancer cells, suggesting the role of cytokines in cancer cell evasion to drug-induced cell death (82–84). Several studies show IL-6 and IL-8 secretion by cancer cells promote multiple drug resistance possibly *via* the autocrine induction of cancer stem-like cells (7) while inhibiting these cytokines can re-sensitize the tumor against the chemotherapeutic drugs (85, 86). Importantly, these studies thus suggest the prognostic values of cytokines to monitor the development of anti-cancer drug resistance.

Secretion of IL-4 and IL-10 by cancer cells has been shown to confer drug-induced resistance, by specifically inhibiting apoptotic pathways (87–89). IL-13 has also been shown to promote drug resistance in lymphoma, although the mechanism is unclear (90). These cytokines can thus be useful as predictive biomarkers, and to monitor resistance towards anti-cancer drugs. Furthermore, in renal cell carcinoma, the cytokines TNF and MMP-9 were found to be useful as a predictive biomarker for the activity of the anti-cancer drug sunitinib activity (91). IL-8 has been shown to confer sunitinib resistance, thus this cytokine can also be used as a predictive biomarker for sunitinib efficacy (92). Another study showed the predictive value of circulating IL-6 and IL1-β for gemcitabine efficacy to treat pancreatic cancer (93). In triple-negative breast cancer and ovarian cancer, TGF-β induces paclitaxel resistance, and TGF-β level measurements could be used to predict responses to chemotherapy drugs (94, 95). Moreover, the levels of circulating cytokines, IL-1α, IL-2, and IFN-α2 are useful to monitor severe, potentially life-threatening immune-related toxicity in response to the use of checkpoint inhibitors (96). Circulating CCL-27 has also been proposed to identify patients with muscle-invasive bladder cancer that will respond to Bacillus Calmette-Guerin (BCG) treatment (97).

## Cytokines and Cancer-Specific Biomarkers for Diverse Cancer Types and Stages, and Their Potential Use at Clinics

With their involvement in immune response as well as cancer development, cytokines have great potential to be used to detect the presence of cancer and to monitor its subsequent severity, including during drug intervention (**Figure 2**). The production of tumor-elicited cytokines in cancer started at TME, which then spread into the circulation. Dysregulation in circulating cytokine levels has been correlated with the presence of many types of cancers, the severity of cancer as well as the effectiveness of a certain therapy thus may serve as quality biomarkers to support diagnosis and prognosis (**Figure 2**). For example in renal cell carcinoma, a high IL-6 correlates to cancer metastasis, while high IL-6 and IL-17 measurements predict cancer recurrence following radical treatment (98). Other recent examples of the use of circulating systemic protein for prognosis are serum CRP levels, in which high levels are associated with poor survival in patients with gastric cancer based on a meta-analysis, independent of country of origin, cancer stage, and study design (99), and serum CCL-2 levels, in which high levels are associated with poor prognosis and survival of patients with pancreatic cancer, independent of gender, age and the stage of cancer (100).

There is, however, functional redundancy of cytokines in diverse pathways, making individual cytokine measurements not specific enough for a certain disease. Several studies used multiple cytokine measurements for cancer diagnosis and prognosis. For example Kawaguchi et al. (101) shows 3 separate cytokine groups that could subtype breast cancers. They also showed a group of circulating cytokines that can identify metastatic breast cancer patients. Similarly,Wang et al. (102) and Semesiuk et al. (103) showed that measurements of a group of cytokines can serve as potential biomarkers to predict metastasis in breast cancers. Additionally, combined analysis of cytokines and their soluble receptors may improve the predictive capacity of the cytokines. Pilskog et al, for example, have shown the predictive value of basal IL-6 in response to sunitinib, while sIL-6 receptor, the receptor of IL-6 that mediate the trans-signaling of IL6, added the prediction of the length of progression-free survival for people with clear cell renal cell carcinoma (104).

Several studies have strategized combining the analyses of circulating cytokines with the circulating cancer-specific proteins to provide more accurate detection of cancer, especially at earlier stages. For example Li et al. (105), combined IL-6, IL-8, and TNF measurements with carcinoembryonic antigen (CEA) and cancer antigen (CA)-724, to improve the screening power of these two tumor markers, as detection rate at early stages of gastric cancer is still very low. In pancreatic ductal adenocarcinomas (PDAC), CA-19-9, the pancreatic cancer-specific protein alone shows moderate diagnostic accuracy with an area under the curve (AUC) of 0.807 to distinguish between PDAC and benign controls, while in combination with cytokine CXCL-10, gives a more accurate diagnostic value with AUC of 0.977 (106). Additionally Kampan et al. (107) show that combined IL-6 with conventional CA-125 cancer marker provides a more accurate diagnosis for high-grade serious ovarian cancer.

## Dysregulated Cytokine Signaling Signature From Peripheral Immune Cells as Cancer Biomarkers

The dysregulated circulating cytokines are not the only immune biomarkers that can be detected in the circulation during cancer. Instead, in cancer patients, peripheral immune cells have dysregulated immune cytokine signaling signatures (**Figure 2**). Recent studies (108, 109) show dysregulated IL-6 secretion from peripheral T cells and dysregulated IFN-γ secretion from peripheral monocytes, even from patients with only localized tumors. The dysregulated cytokine signaling signatures in the immune cells strongly predict the risk of future relapse in two independent breast cancer cohorts, which point to systemic cytokine responsiveness as biomarkers to evaluate immune status in breast cancer patients. Additionally, the immune signature of peripheral T cells, analyzed by phosphorylated-STAT, can differentiate colorectal cancer patients from the healthy controls with an AUC of 0.94 (110). These studies thus point to the use of the cytokine signaling signatures of peripheral immune cells as an alternative venue for cancer diagnosis and prognosis.

## The Established and Emerging Devices for Cytokine and Other Cancer-Specific Biomarker Detections and Their Potential Technological Challenges

Recently, there have been technological advancements and innovative approaches to study and measure multiple cytokines utilizing a minimum amount of blood samples. Below we will discuss the current and emerging technologies (**Table 1**) that could be used to detect circulating cytokines and secreted signatures of cytokines from peripheral cells, with multiplexing capacity that not only provide a high level of specificity, but also sensitivity to support cancer diagnosis and prognosis. We will further discuss the technological advancement required to improve the quantification of the cytokines and other circulating protein biomarkers in liquid biopsies.

**Table 1:** Established and emerging devices for cytokine detection.

Platform	Deetection Range	Sample used	References
**Label-based immunoassays**			
a. ELISA	pg/ml	Serum/Plasma	(3, 111)
b. 125I-streptavidin immunoassay	fg/ml	Serum/Plasma	(112)
c. Fluorescent-based immunoassay	pg/ml	Serum/Plasma	(113)
d. Immuno-PCR	fg/ml	Serum/Plasma	(114–117)
e. Immuno-DNA	fg/ml	Serum/Plasma	(118)
f. Nanoparticle-labeled immunoassays	pg/ml	Serum/Plasma	(119, 120)
g. Aptamer assays	pg/ml	Serum/Plasma	(121–123)
h. Imprinted polymer assays	pg/ml	Serum/Plasma	(124, 125)
**Multiplex label-based immunoassays**			
a. Protein microarrays	pg/ml	Serum/Plasma	(126–128)
b. Bead-based flow cytometry	pg/ml	Serum/Plasma	(129)
c. Luminex	pg/ml	Serum/Plasma	(130–135)
d. SiMoA	fg/ml	Serum/Plasma	(136–138)
e. Immunoaffinity chromatography	pg/ml	Serum/Plasma	(139–141)
f. Hydrogel microparticle with microfluidic system	pg/ml	Serum/Plasma	(113)
**Non-antibody based**			
a. Classical mass spectroscopy	pg/ml	Serum/Plasma	(142–144)
b. Targeted mass spectrometry	pg/ml	Serum/Plasma	(145, 146)
c. Affinity mass spectrometry	pg/ml	Serum/Plasma	(147, 148)
**Label free**			
a. Electrochemical biosensors	pg/ml	Serum/Plasma	(149, 150)
b. Electro chemiluminescent	pg/ml	Serum/Plasma	(151, 152)
c. Electro chemiluminescent with microfluidic system	fg/ml	Serum/Plasma	(153)
d. Electrochemical aptamers	pg/ml	Serum/Plasma	(154)
e. Nanoparticle-based electrochemical aptamers	fg/ml	Serum/Plasma	(155)
f. Surface plasmon resonance (SPR)	pg/ml	Serum/Plasma	(156–158)
g. Localized SPR	fg/ml	Serum/Plasma	(159–164)
**Single cell secretion assays**			
a. ELISpot	–	Cells	(165)
b. FluoroSpot	–	Cells	(165)
c. Microfluidic with labelled biosensor	ng/ml	Cells	(166–169)
d. Microfluidic with label-free biosensor	pg/ml	Cells	(170)

### Label-Based Immunoassays

The most widely used label-based immunoassay to quantify soluble protein in liquid biopsies such as serum and plasma is the Enzyme-linked Immunosorbent Assay (ELISA) (3). ELISA platforms are widely available, making them easily accessible to be used as diagnostic and prognostic tools for patients in various demographic areas. The sandwich-type of ELISA is more specific and reproducible than the competitive ELISA, as the former requires two specific bindings of antibodies to different epitopes of the soluble proteins being measured. One antibody serves as the solid support for the assay, and the other is conjugated with reporter molecules, originally enzymes, but now other types of reporter molecules are also available, which will determine the platform used for the readout. Competitive ELISA on the other hand, measures the concentration of protein of interest, by detecting the amount of competitive reference antigen that binds to the specific antibodies after the binding of the protein of interest to the specific antibodies has occurred. Currently, many commercial ELISA kits are based on the classical colorimetric assay and use a standard absorbance-based plate reader as the readout. These kits commonly use an enzyme-based reporter molecule, such as biotin coupled with a streptavidin-horseradish peroxidase (HRP) conjugate and addition of a chemiluminescent substrate (TMB), in which many of them offer a lower limit of detection (LoD) in the low pg/mL concentrations (111).

As cytokines, cytokine receptors and cancer-related circulating proteins are commonly present below pg/mL concentrations, improvements of this classical ELISA to lower the LoD into the fg/mL or even single molecule levels are desired. One strategy is to use a much more sensitive reporter molecule. Indeed, Drukier et al. (112) has employed ^125^I-streptavidin as the reporter molecule and developed a multiphoton method to detect the molecule. This strategy has successfully increased the sensitivity of conventional ELISA by 200-1000 folds into the fg/mL. Recently, Cesaro-Tadic et al. (113) have used a microfluidic system and employed a fluorescent tag as the reporter molecule to measure TNF and obtained LOD of 20 pg/mL.

Another strategy is using a DNA sequence as the reporter molecule, with DNA amplification as a means to detect the amount of protein of interest captured by the DNA-labelled antibodies. The DNA sequences could be linked to the antibodies using either a step-wise assembly of biotinylated antibody, streptavidin, and biotinylated DNA, an assembly of a biotinylated antibody, and an antibiotic-DNA conjugate, or a direct synthesis of antibody-DNA conjugate (114). The immuno-PCR method is capable of providing up to 10^9^ increase in detection sensitivity in comparison to the classical ELISA, after the advancements in the production of the DNA-labelled antibodies, assay formats, and readout methods, as reviewed in (115). Furthermore, gold nanoparticles have also been used to attach multiple DNA sequences to the antibodies, allowing for detection without DNA amplification (117). Nam et al. (118) for example have developed a gold nanoparticle-based colorimetric DNA detection, that allows simple and straightforward detection of IL-2 with 0.45 fg/mL of LOD was reported. Proximity ligation assay and proximity extension assay are other ways of detecting the reporter DNA in the immuno-PCR methodology. Two separate antigen-specific antibodies are used to detect the same protein of interest in a complex biological liquid. The two antibodies are conjugated with DNA sequences that form amplifiable sequences by ligation or by DNA polymerase extension when they are in close proximity. Gullberg et al. (119) have used this technology to detect IL-2 and other cytokines with 0.015 fg/mL of LOD. Schallmeiner et al. (120) used three instead of two antibody-DNA conjugates to detect as little as a hundred molecules of VEGF and other cancer-related proteins from biological liquids. Ke et al. (116) used digital single-molecule detection of the DNA instead of the quantitative PCR to improve the precision and detection sensitivity of this technology.

Aptamers can also be used instead of antibodies to bind to specific target molecules. They are a single nucleic acid strand of either DNA or RNA that are obtained using a procedure called systemic evolution of ligands by exponential enrichment (SELEX) coined by the Gold lab in 1990 (121). They have been used also for the detection of cytokines (122) including both, ELISA and immuno-PCR approaches (123).

Molecularly imprinted polymers have been used as the surrogate of the capture antibody. In this technology, the polymer template is designed to have cavities with a specific shape that could capture specific cytokines. The antigen-specific detection antibodies with fluorescent tags then are applied. Using the system, Deng et al. (124) developed a reusable molecular imprint polymer to detect IL-1β with a LOD of 10.2 pg/mL and could reuse the biosensing device more than three times with a coefficient of variation of 2.08%. Tao et al. (125) used molecular imprint containing luminescent reporter molecules, to directly quantify the bound IL-1 from plasma using the biosensor alone which gave 2 pg/mL LOD and >95% reversibility of the platform, even after being used more than 25 times.

### Multiplexed Label-Based Immunoassays

Since cytokines commonly exert their effects as part of an overall signaling network, there are increasing interests in analyzing more than one cytokine from liquid biopsies. Indeed, the concept of the sandwich ELISA has been used to rapidly develop the multiplexing capability of immunoassays in the last few years, which has been extensively reviewed in (129). The multiplexed sandwich ELISA came in planar- and microbead-based arrays. The protein microarrays are planar-based arrays with antibodies being placed on the solid surface of the array chip. The first planar-based array was developed in 2001 to measure TNF, IFN-α, IFN-γ, IL-1α, IL-1β, IL-6, and IL-10 from a single sample of culture media of stimulated THP-1 cells. Here, cytokines detected by the array of antibodies are detected in pg/mL using HRP/TMB (130). The recent advancement in this planar technology is the measurement of 80 distinct cytokines from tear fluid (126). Recently a digital protein microarray has been developed to monitor the critically ill COVID-19 patients from having cytokine storms, with the technology provides rapid daily cytokine assays at clinics (127). It uses a fluorescence optical scanner to detect IL-6, TNF, IL-1β, and IL10 with a high sensitivity of <0.4 pg/ml.

Another popular approach of multiplexing in cytokine measurement is using bead microspheres of around 5-7 micrometer in diameter as the base of the immunoassay. These beads are uniquely colored and conjugated with specific capture antibodies, allowing the differentiation of beads that capture different target proteins. The captured proteins from a liquid biopsy sample will directly bind to another protein-specific antibody conjugated with reporters such as fluorophores. This multi-protein profiling will not only provide the list of cytokines that are present, but also the amount of the cytokines in the liquid biopsy. The analysis of the beads can be done using a particle-based flow cytometry (128) that has developed into Luminex xMAP technology in 1997 (129). This technology can perform up to 500 bioassays simultaneously from a small quantity of liquid biopsy. Using conventional flow cytometry, BD cytometric bead array could detect 30 proteins in solution simultaneously. The bead-based systems detect cytokines from the liquid biopsy with sensitivity in the low pg/mL. Several studies have shown that the combination of microfluidic-based detection technology and the bead-based immunoassay has resulted in multiplexing of the cytokine detection with sensitivity in the low pg/mL (131–134). Bead-based immunoassay has also been combined with digital Single Molecule Counting detection, in which single-molecule fluorescent signals are counted using a laser digital counter. Multiplex detection of IL-6, IL-4, and IL-10 using this method in plasma resulted in pg/mL sensitivity (135). Another approach is a single molecule array (SiMoA) that places each bead into a well before digitally analyzed the fluorescence (136). Multiplexing cytokine measurement with this method has given sensitivity up to astonishing low fg/mL concentration (137, 138).

A different format of multiplex immunoassay is recycling immunoaffinity chromatography. This format is only a single antigen-specific antibody system. The capture antibodies are immobilized in glass beads packed in capillary immunoaffinity columns. The cytokines in a biological sample are labeled with fluorescence dyes and then captured by the antibodies in the capillary system. The LOD of this technology for cytokines has been shown in pg/mL concentrations, and the bead-based antibody platform could be reused up to 200 times (139). Castle et al. (140) have developed this recycling system to analyze 24 cytokines from plasma and cervical fluid that gives a LOD of 2 pg/mL and highly reproducible results. This technology has formed the lateral flow immunochromatographic assay or also known as the rapid test for home, laboratory, and point of care testing without the need for specialized equipment. With a modification of the immunoassay format, Worsley et al. (141) have used a lateral flow assay format utilizing two distinct fluorescent beads/microspheres to detect IL-6 and TNF simultaneously in plasma with LOD of 7.15 pg/mL and 10.7 pg/mL respectively. Appleyard et al. (113) used a range of hydrogel microparticles to analyze IL-2, IL-4, and TNF in complex media, simultaneously using high-velocity microfluidic scanning and reached LOD between 1-8 pg/ml.

Despite the popularity of the multiplexing assays, only a few have been validated for IVD applications (129). Many technical challenges have been associated with multiplexing in both planar and microsphere formats, including selection and specificity of antibodies and interference between antibodies, calibration procedure, the range of linearity, inter-and intra-assay variations, as well as limitation in detection precision, quantification limits, and reference measures. These challenges have hindered the use of many multiplexed cytokine assays in clinical settings. Thus, guideline in assessing analytical multiplex assay performance needs to be established such as for analytical validation and quality control, before being used at clinics (163). In a large collaborative international study, twelve laboratories compared various immunoassays including ELISA, Luminex, and chemiluminescence assays to measure human cytokines, IL-1β and IL-6. The intra-laboratory variations were within expected values, while Luminex showed the lowest inter-laboratory variations (164).

### Mass Spectrometry

While label-based immunoassays have been commonly used to measure circulating cytokines, mass spectroscopy (MS) has becoming increasingly attractive to analyze proteins in biological fluids and has gained preference for diagnostic applications, as reviewed in (171). Not only this method does not depend on target-specific antibodies, but it is also capable of identifying different isoforms of a certain protein or its post-translational modifications, that may serve unique biological functions and thus have distinct predictive values for diagnosis and prognosis. MS measures the mass to charge ratio of ions in a liquid sample. MS analysis will usually result in a spectrum plot of mass-to-charge ratios against the detected intensity of the ion signals. Measurement of multiple cytokines or secretomes that are released by cells, such as monocytes with 200 proteins (172) and macrophages with 775 proteins (173) being detected in serum-free media, have been successfully done using MS with sensitivity in low picogram. The challenge with MS is that the detection can be masked by matrix interferences and signal suppression, exemplified by a LOD of only in low ng/ml even after spiking of the biological complex fluid such as serum (174). Currently, for complex biological liquids, technology advancements of MS techniques have been proposed and resulted in highly sensitive and specific detection of certain proteins and their isoforms from the circulation. These include tandem MS and affinity capture MS.

The tandem MS combines two or more mass analyzers using a reaction step to increase the MS ability to analyze chemical samples. Usually, the first analyzer will fragment the target ions to produce a range of smaller ions, then the consecutive analyzers will analyze only the fragmented ions from the specific target ions. This method will ignore non-target ions that flow into MS, improving the specificity whilst maintaining detection accuracy. The targeted MS is a variation of tandem MS, in which the consecutive analyzers will only analyze the specific fragmented ion of interest increasing precision and sensitivity. The successful use of targeted MS and the targeted MS are exemplified by its use in clinical settings to measure hard-to-detect low-molecular-weight hepcidin and its isoforms from biological fluids of cancer patients (142, 143). Affinity-capture MS has also gained popularity. The technique involved affinity capture, using antibodies or aptamers for example that recognizes specifically the protein of interest from complex biological liquids before the samples are subjected to the mass analyzers. The strategy has been used not only to quantitate hard-to-protein such as hepcidin but also to differentiate between its isoforms (144). For example, using affinity capture MS, Nedelkov et al. (145) quantifies with precision not only the human endogenous insulin, but also most of the therapeutic insulin analogs, all with sensitivity in the low pg/mL concentrations. This is useful to test the cause of hypoglycemia or to detect insulin doping.

MS method has started to gain trust in cancer proteomic analyses as improved standardization of sample preparation, MS techniques, the statistical evaluation of the data and the reporting or publishing of the study are developing (171, 175). Many of the standardized MS measurements have been now used in clinical diagnostics, including C-peptide, insulin-like growth factor-1, angiotensin 1 among others as reviewed in (171).

### Label Free Biosensing

Label-free biosensors are taking immunoassays a step forward in the diagnostic field. They rely on the combination of specific bioreceptors with transducing elements that detect in real-time the presence of analytes in the liquid samples (176). This interaction produces a readily measurable signal that can be detected due to changes in current (electrochemical), mass (piezoelectric), or the properties of light (optical), proportional to their concentration in the sample. This capability makes them very attractive analytical tools since they can achieve high sensitivities without requiring the use of any secondary fluorescent labels or amplification steps, promoting a more reliable reading while saving time and reducing costs. The ease of use of label-free biosensors has sparked a flood of analytical solutions for cytokine detection. Here we present some examples of cytokine detection based on electrochemical and optical-based label-free biosensors.

Electrochemical biosensors are based on two electrodes where cytokines can be detected using reporter molecules that produce electroactive substrates that change the properties of the electric field. They have been employed for TNF detection using either polyguanine-functionalized silica nanoparticles (146) or alkaline phosphatase-functionalized gold nanospheres (147) as electroactive substrates, achieving LODs of 5 pg/mL and 10 pg/mL respectively. A variant of electrochemical measurement is the electrochemiluminescent technique in which luminescence is produced by an electrochemical reaction in a solution. In particular, Sardesai et al. (148) have developed a novel electrochemiluminescence immunosensors featuring a capture-antibody-decorated single-wall carbon nanotube to measure IL-6 in serum that gave LOD of 0.25 pg/mL. When combined with microfluidic technology, serum IL-6 was detected with LOD of 10 fg/mL (177). Using electrochemiluminescence approaches, another group has reported TNF detection with LOD of 7 pg/mL (178). This type of label-free biosensors can also employ aptamers as the biorecognition element. Liu et al. (149) have used electrochemical DNA aptamer-based biosensor to detect IFNg in high pg/mL concentrations, while Li et al. (150) have used gold-nanoparticle modified DNA aptamer to detect platelet-derived growth factor β-chain homodimer (PDFG-BB) at 1.9 fg/mL concentrations. Additionally, aptamers for cytokines have been proposed not only for diagnostic but also for anti-cytokine therapeutic purposes (179).

Label-free optical biosensors do not require the use of any reporter molecules to detect the cytokines present in the sample. This type of biosensor can detect minute changes in the properties of light (intensity, refractive index, resonance, or wavelength) produced by cytokine interaction with a biological receptor attached to the plasmonic or silicon photonic transducer (180). This makes these sensors advantageous over electrochemical biosensors, since they are more stable to changes in pH or ionic concentration, making them easier to operate (181). Surface plasmon resonance (SPR) biosensor is the most widely used label-free optical biosensor in the biomedical field. It generates an electromagnetic wave (plasmon) at the interface between a metal (i.e. gold) and a dielectric medium by light excitation and is sensitive to the biological interactions occurring close to the metal surface. Thus, SPR offers a perfect opportunity to detect analytes in real-time and free of labels and has been employed for the detection of different cytokines, including IL-6 (151), IFN-γ (153) and TNF (152), achieving LODs in the pg/mL-ng/mL range. Nanotechnology has promoted the field of nanoplasmonics, which employs arrays of nanostructures including nanorods, nanostars, nanodisks, and nanoholes that, when excited with light, exhibit confined electromagnetic fields such as in localized SPR (LSPR) or enhanced extraordinary optical transmission effects (EOT). This is translated into more compact platforms with easier light-excitation methods that can be multiplexed for multiple cytokine detections. Several works have presented improved sensitivity of the detection of cytokines in complex biological fluids utilizing LSPR nanostructures or EOT nanohole arrays, which include detection of IL-1β, IL-18, TNF, and MMP-3 with LOD in low pg/mL and fg/mL (154–156, 182–184). The opportunities offered by these label-free optical biosensors are numerous since these devices can adopt a great variety of bioreceptors besides antibodies, including aptamers (185) and triplex-forming DNA probes (165, 185) through different chemistry approaches (166) which can improve the detection of multiple cytokines, as well as enrich the analysis with other genetic and epigenetic biomarkers (167, 168). Other label-free optical biosensors based on silicon photonics, such as micro-ring resonators, have been used for the simultaneous detection of pg/mL concentration of cytokines, including IL-2, IL-4, IL-5, and TNF (169). These types of optical biosensors employ waveguides structures where light can propagate and can be controlled with higher precision by the use of sophisticated signal interrogation approaches (170) that can improve further the sensitivity, robustness and reliability of the cytokine detection.

### Detection of Cytokine Secretion From Single Cells Using Label-Based and Label-Free Approaches

Dysregulated cytokine secretion profile of the peripheral immune cells has been proposed to be an alternative cancer detection biomarker (**Figure 2**) studies (108, 109) 94 (110), and could be explored using the different label-based and label-free approaches explained above. The detection of the secreted cytokines from a single cell could be performed indirectly using flow cytometry, by blocking the secretion of the proteins and measure the intracellular cytokine levels within the cells. The enzyme-linked immune spot assays (ELISpot) and the variant FluoroSpot have been used to directly detect cells that are capable of secreting certain cytokines following activation, however, this technique does not provide the amount of cytokines secreted from a single cell (157). Several new technologies have been developed to directly measure cytokine secretion from a single cell. Microfluidic devices capable of capturing specific single cells followed by detection of multiple cytokines using specific antibodies and various biosensors have been developed (158–161), that could currently detect the cytokines in ng/mL range. Recently, Li et al. have successfully developed such technology utilizing label-free optical biosensors based on nanoplasmonics to detect cytokine secretion from a single cell, reaching LODs in pg/mL range in real-time (162). They were able to do that from a very low volume of sample (180 nL) using a unique microfluidic device that allowed to isolate the single cell in a specialized compartment that kept the appropriate conditions such as humidity and avoided sample evaporation. This allowed to keep the cell alive for up to 72 hours and facilitated the sensitivity detection required to achieve single-cell resolution.

### Sample Handling and Other Improvement Required for the Applications of Circulating Cytokine Measurements for Diagnosis and Prognosis Strategy in Clinical Settings

The use of a minimally invasive liquid biopsy for the detection of cancers is a promising strategy to provide diagnostic tools at the point of care. However, before a cytokine measurement methodology can be used at clinics, standardization of the methodology itself needs to be in place to provide an accurate diagnosis. Sample handling is the key to perform a reliable quantitative analysis. For any quantitative analysis, it is important to have a representative and sufficiently homogeneous sample collected for the analysis. Moreover, storage conditions between sampling and analysis need to be properly controlled to ensure there is no loss of analytes due to degradation. Further, calibration using standards to measure the range of analyte levels and quality controls to measure intra- and inter-assay variations need to be in place. Standardization of the measurement between different laboratories especially when employing different technologies needs to be assessed before specific cut-off values can be determined for specific analytes in a specific disease. For example, the standardization of the cytokine flow cytometry method has achieved a good interlaboratory precision, which will allow precision when using cytokine signatures of peripheral immune cells as diagnostic biomarkers. Another example of standardization is the use of an algorithm for ovarian cancer diagnosis, including RMI (Risk of Malignancy Index) and ROMA (Risk of Ovarian Malignancy Algorithm) to improve the inherent characteristics of CA125 and HE4 ovarian cancer biomarkers (186). In this regard, research in microfluidic devices is focused toward the goal of standardizing the analyses and minimizing human errors, and different strategies have been already demonstrated, including fluid handling automation (187) and sample purification approaches (188) that could be easily coupled to the different analytical tools discussed in this review to monitor cytokine concentrations more efficiently.

## Conclusion

Cytokines are produced by diverse types of cells and commonly mediate intercellular communication within the TME to support cancer development. Accumulating data show that a cytokine storm created within the TME is responsible for cancer formation, metastasis, and further drug resistance. Additionally, cancer cells also produce cancer-specific proteins that potentially play a role in cancer survival, growth, metastasis, and recurrence. On the other hand, there are also cytokine receptors that can either positively or negatively regulate cytokine activity. The soluble form of the receptors is common for many cytokines as part of the homeostatic process to prevent cytokine storms. Detection of circulating cytokines, soluble cytokine receptors, and cancer-related proteins in the circulation could help provide cost-effective and accessible diagnosis and prognosis of cancer and the treatment outcome.

Cytokine, cytokine receptor, and cancer-specific protein quantification in the circulation hold the potential application for cancer diagnosis and prognosis and have become the current forefront research topics in cancer. This is because they are relatively easy to measure and could provide a non-invasive alternative for diagnosis, especially for cancers in which biopsy approaches are contraindicated, such as glioma. Additionally, since cytokines mostly work in a network system at a certain time and place, measurement of multiple cytokines from a single sample might be necessary for accurate diagnosis and prognosis. Currently, some technologies could be used to measure multiple cytokines from a small quantity of biological fluids to provide a minimally invasive diagnostic procedure. However, several requirements, especially standardization of sample handling and measurement procedures need to be addressed before the test could be brought into clinical settings.

## Author Contributions

AK and MP concepted the manuscript. AK and CH drafted the whole manuscript. AK, CH, AM, and MP critically read the manuscript. All authors contributed to the article and approved the submitted version.

## Conflict of Interest

The authors declare that the research was conducted in the absence of any commercial or financial relationships that could be construed as a potential conflict of interest.
